# Retrospective external validation of the Mayo Delirium Prediction tool in a Swiss cohort of medical and surgical inpatients

**DOI:** 10.1186/s12911-026-03660-5

**Published:** 2026-06-29

**Authors:** Michela Venturini, Nayeli Schmutz Gelsomino, Sandeep R. Pagali, Martin Zozman, Felix Buddeberg, Mary-Anne Kedda, Marius Möller, Simone Pascale Wildhaber, Reto Stocker, Benjamin T. Dodsworth

**Affiliations:** 1 PIPRA AG, Zurich, Switzerland; 2 Department of Clinical Research, University of Basel, Basel, Switzerland; 3 Department of Medicine, Mayo Clinic, Rochester, MN USA; 4 Kusnacht Practice AG, Zurich Zollikon, Switzerland; 5 Hirslanden Klinik Hirslanden, Zurich, Switzerland

**Keywords:** Delirium prediction, External validation, Model generalisability

## Abstract

**Background:**

Delirium is a frequent complication in hospitalised patients, associated with long-term cognitive impairment, prolonged hospital stays, and increased mortality. Early risk assessment is essential for implementing preventive strategies. The Mayo Delirium Prediction (MDP) tool, developed using data from a large academic hospital in the United States, includes separate models for medical and surgical patients. The MDP is available in its original and recalibrated versions. However, its performance in non-US population remains unknown. The objective of this study is to externally validate the MDP tool in a cohort of hospitalised patients from a Swiss private hospital.

**Methods:**

This retrospective validation study used routinely collected clinical data from adult medical and surgical inpatients admitted to a Swiss hospital in May and June 2023. Delirium diagnosis was based on the Delirium Observation Screening Scale (DOSS). Predictive performance was assessed using the Area Under the Receiver Operating Characteristic curve (AUROC) and calibration plots. Additional sensitivity analyses were performed to assess the model’s robustness and mitigate possible biases in the evaluation.

**Results:**

The original medical MDP tool achieved the best predictive performance in the external validation cohort of 947 patients, with an AUROC of 0·87 (95% CI: 0·85–0·90). The original surgical MDP tool showed relatively lower performance in the external validation cohort of 1212 patients, with an AUROC of 0·73 (95% CI: 0·69–0·78). Performance remained acceptable across all sensitivity analyses, with only a modest reduction in AUROC. The original MDP tool consistently outperformed recalibrated versions across all scenarios.

**Conclusions:**

The MDP tool, particularly the medical model, maintained strong predictive performance in an independent setting. These findings support its generalisability and potential integration into routine clinical workflows for delirium prevention.

**Supplementary Information:**

The online version contains supplementary material available at https://doi.org/10.1186/s12911-026-03660-5.

## Background

Delirium is a common condition among hospitalised patients, with an incidence ranging from 14% to 32% in older surgical patients, depending on the type of surgery [1], and approximately 13·5% in older medical patients [2]. Its onset is associated with poor outcomes such as long-term cognitive impairment [3], prolonged hospital stay [4], and increased mortality [5]. A range of pharmacological and non-pharmacological strategies have demonstrated efficacy in preventing delirium [6]; however, early identification of patients at risk is critical to enable their effective implementation and improve clinical outcomes. Several delirium predictive tools have been developed to facilitate early identification [7, 8]; nonetheless, limitations in study design, data collection, and the lack of external validation limit their generalisability across diverse patient populations and healthcare settings.

The Mayo Delirium Prediction (MDP) tool [9] was developed at Mayo Clinic Hospital, Rochester, Minnesota, with the aim of introducing an accurate prediction model derived from a large and heterogeneous patient population. It includes two distinct models designed to predict delirium onset in medical and surgical patients. Initially derived from a retrospective cohort of 80,000 patients, the MDP tool demonstrated good predictive performance, with an Area Under the Receiver Operating Characteristic curve (AUROC) of 0·84 (95% CI: 0·83–0·85) in the internal validation cohort. The tool was subsequently modified to adjust for fall risk as binary rather than a continuous predictor, facilitating its integration with different fall risk scores. This adaptation, followed by a recalibration on a new patient cohort from the same hospital, resulted in a simplified version of the MDP tool, which maintained comparable performance, with an AUROC of 0·82 (95% CI: 0·81–0·83) [10]. The recalibration was performed on a smaller cohort and with a different diagnostic approach for delirium but was intended for the same clinical context of the original tool.

Although the MDP tool has demonstrated satisfactory predictive performance, all evaluations to date have been conducted using US population data. External validation across different hospital settings is therefore fundamental to assess its generalisability and to support its adoption in clinical practice. This study aims to externally validate the MDP tool in a cohort of medical and surgical patients admitted to a Swiss private hospital.

## Methods

### Study design and validation cohort

Data used in this validation study were collected for a quality improvement project (QIP) from a Swiss, private 335-bed hospital. The project included all medical and surgical inpatients aged 60 or older, admitted between May 1, 2023, and June 30, 2023 [11]. The QIP received a waiver Req-2023-00307 from the Zurich Cantonal Ethics committee.

### Comparison of original and validation cohorts

The development, recalibration, and validation datasets were each derived from separate single-centre patient cohorts. The development and recalibration cohorts were collected retrospectively from Mayo Clinic Hospital, Rochester, Minnesota. In contrast, the validation cohort was collected prospectively. The inclusion criteria of development and recalibration cohorts were slightly different than the ones of the validation cohort. The development cohort included patients aged 50 or older, admitted between January 1, 2012, and December 31, 2017. Patients admitted with acute substance use disorders (intoxication or withdrawal) as their primary diagnosis were excluded. The recalibration cohort comprised patients meeting the same inclusion criteria, admitted between December 19, 2019, and June 12, 2020.

Fall risk was included as a predictor in the MDP tool. In the development cohort the Hendrich II Fall Risk Model [12] was available, whereas the recalibration cohort included the Hester Davis Scale [13] given local practice changes. In this study an approximation of both scores were included, in addition to the ePA-AC [14] nursing assessment fall risk. Details about the risk scores are available in the appendix.

Delirium diagnosis was obtained using a different tool in each cohort. In the development cohort, delirium diagnosis was obtained either from physician documentation or from nursing documentation of Confusion Assessment Method for the Intensive Care Unit (CAM-ICU) [15] assessments, both for ICU and non-ICU patients. In the recalibration cohort, following an internal QIP project that focused on improved delirium detection [16], delirium was defined as a positive CAM assessment documented by nursing staff: CAM-ICU for ICU patients and DTS/brief-CAM [17] for non-ICU patients. In this validation cohort, delirium diagnosis was defined as either a Delirium Observation Screening Scale (DOSS) [18] score of three or above, or the presence of an ICD-10 diagnosis code for delirium during the hospital stay. Both CAM-ICU and DTS/brief-CAM were derived from the Confusion Assessment Method (CAM) [19]. CAM-ICU was specifically designed for critically ill patients, including those who are non-verbal or mechanically ventilated. In contrast, DTS/brief-CAM was developed for non-ICU patients who are alert or only mildly impaired, particularly in emergency departments or general hospital wards. The DOSS was developed for use in medical wards, geriatric, and psychiatric units. While the CAM-derived tools are intended to detect delirium directly and produce a binary outcome, the DOSS is an observation-based scale (ranging from zero to 13) used to monitor signs of delirium over time.

### Predictors

Collected predictors included demographic information, admission type, comorbidities, laboratory measurements, risk scores, and medication data. The Hendrich II Fall Risk Model and the Hester Davis Scale were reconstructed using routinely collected clinical variables [see Additional file 1]. Predictors were not collected specifically for delirium risk assessment but were part of standard care. Information on race or ethnicity was not available, as these variables were not routinely collected. The list of predictors is available in the appendix [see Table S7, Additional file 1]. All predictors were reviewed and harmonised; creatinine values originally recorded in µmol/L were converted to mg/dL, and haemoglobin values recorded in g/L were converted to g/dL.

### Outcome

The outcome predicted by the MDP tools is the onset of delirium. In both the development and recalibration studies, delirium onset could occur at any point during the hospital stay. In the validation cohort, for surgical patients, delirium was assessed up to seven days following surgery or until discharge, whichever occurred first. For medical patients, delirium was assessed until discharge. Nurses were not informed about the validation project and were instructed to perform the DOSS on all patients as part of the QIP.

### Sample size

The sample size was not formally calculated, as the data was obtained as part of a QIP. The precision of model performance estimates was assessed retrospectively using the method proposed by Hanley and McNeil [20]. For the medical cohort (947 subjects; delirium incidence of 23%) and a targeted AUROC of 0·75, the estimated 95% CI width for the AUROC was 0·08. For the surgical cohort (1212 subjects; POD incidence of 12%), the corresponding width was 0·10. This precision was deemed sufficient to provide meaningful performance estimates.

### Missing data

Only predictors included in the models were imputed. Binary predictors were imputed using the mode from the development cohort. The Charlson Comorbidity Index and reconstructed Hester Davis Scale had no missing values. The reconstructed Hendrich II Fall Risk Model had one missing value, imputed using the median from the validation cohort because its reconstructed version might have differed from that in the development data. The ePA-AC fall risk was not imputed, as it was used in the sensitivity analysis.

### Statistical analysis

Cohort characteristics were reported overall and stratified by delirium onset. The validation cohort was also compared with the development and recalibration populations from the previous studies. Categorical variables were presented as n (%), and continuous variables as median (IQR). The Kruskal–Wallis test was used for continuous variables, while the χ^2^ test for categorical variables. Missing data were reported as n (%).

The MDP tool for medical and surgical patients were evaluated separately in terms of predictive performance, in their original and recalibrated versions [see Table S8, Additional file 1]. Reconstructed Hendrich II Fall Risk Model, reconstructed Hester Davis Scale, and ePA-AC fall risk were included in the analysis as continuous variables to validate the original models, and as binary variables for the recalibrated version. The ePA-AC fall risk is categorical with either “no”, “elevated” or “yes” (original: “nein”, “erhöht”, “ja”). Here it was binarized for use in the recalibrated models, combining “elevated” and “yes”, based on the observed relationship between the ePA-AC fall risk and the reconstructed Hendrich II Fall Risk Model and Hester Davis Scale, rather than the outcome of the validation cohort, to preserve the independence of the external validation. Model performance using the ePA-AC fall risk was assessed after excluding all patients with missing values for this predictor.

The reconstruction method for the Hester Davis Scale used diagnosis-related variables collected during hospitalisation without timestamp. In addition, predictors used by the tool such as congestive heart failure and fracture, were also reconstructed using variables without timestamp. These variables carry a risk of information leakage (bleed over). Therefore, a sensitivity analysis was performed with these variables removed or imputed with the mode. Analyses were also repeated after excluding patients assessed for delirium less than once per day.

Model performance was evaluated in terms of discrimination, calibration, and clinical utility. Discrimination was assessed using the AUROC, with confidence intervals calculated via bootstrapping (*n* = 1000). Calibration was evaluated using calibration plots, including subgroup analyses by sex assigned at birth, and age (≤ 75 vs. >75). Decision curve analysis (DCA) was used to assess clinical utility across thresholds. All analyses were performed using Python 3·10·12.

## Results

### Participant characteristics

A total of 5279 patients were admitted during the timeframe of the QIP study period. After excluding patients younger than 60 years and outpatients, 2159 patients remained. The overall population had a median age of 74·0 years (IQR 67·0–81·0), and 44% were female. Patient characteristics, including missing data information, are summarised in Table 1. Patients who experienced delirium were older and had a markedly higher incidence of dementia and a history of delirium, as well as a slightly higher incidence of psychiatric disorders. In addition, they were more likely to have sustained a fracture, and their fall risk scores were higher.

**Table 1:** Cohort characteristics stratified by delirium onset

	Missing	Overall (*n* = 2159)	No delirium (*n* = 1793)	Delirium (*n* = 366)	*p* value
Female	1	953 (44·2%)	788 (44·0%)	165 (45·1%)	1·000
Patient type	0				< 0·001
Medical		947 (43·9%)	730 (40·7%)	217 (59·3%)	
Surgical cardiac		346 (16·0%)	297 (16·6%)	49 (13·4%)	
Surgical non-cardiac		866 (40·1%)	766 (42·7%)	100 (27·3%)	
ICU admission	0	133 (6·2%)	92 (5·1%)	41 (11·2%)	< 0·001
ED admission	0	602 (27·9%)	404 (22·5%)	198 (54·1%)	< 0·001
Dementia	0	115 (5·3%)	0 (0·0%)	115 (31·4%)	< 0·001
CHF	0	234 (10·8%)	178 (9·9%)	56 (15·3%)	0·101
Atrial Fibrillation	0	440 (20·4%)	351 (19·6%)	89 (24·3%)	1·000
History of Delirium	1213	45 (4·8%)	22 (2·8%)	23 (15·8%)	< 0·001
Fracture	0	52 (2·4%)	28 (1·6%)	24 (6·6%)	< 0·001
Ulcer (any stage)	0	52 (2·4%)	35 (2·0%)	17 (4·6%)	0·117
Psychiatric disorder	0	95 (4·4%)	68 (3·8%)	27 (7·4%)	0·106
COPD	0	36 (1·7%)	33 (1·8%)	3 (0·8%)	1·000
Opioid analgesic	0	348 (16·1%)	298 (16·6%)	50 (13·7%)	1·000
Opioid antagonist	0	0 (0·0%)	0 (0·0%)	0 (0·0%)	1·000
Red blood cell transfusion	0	10 (0·5%)	7 (0·4%)	3 (0·8%)	1·000
Hendrich II Fall Risk Model	1	1·0 (1·0–3·0)	1·0 (1·0–2·0)	3·0 (1·0–6·0)	< 0·001
Hester Davis Scale	0	8·0 (5·0–10·0)	7·0 (5·0–10·0)	11·0 (8·0–14·0)	< 0·001
ePA-AC fall risk	354	1·0 (1·0–2·0)	1·0 (1·0–2.0)	2·0 (1·0–2·0)	< 0·001
High fall risk (Hendrich II)	1	257 (12·7%)	141 (7·9%)	134 (36·6%)	< 0·001
High fall risk (Hester Davis)	0	248 (11·5%)	121 (6·7%)	127 (34·7%)	< 0·001
Charlson Comorbidity Index	0	4·0 (3·0–5·0)	4·0 (3·0–5·0)	5·0 (4·0–7·0)	< 0·001
WBC count, 10⁹ cell/L	660	7·5 (6·0–9·5)	7·3 (5·9–9·2)	8·2 (6·5–10·7)	< 0·001
Haemoglobin, g/dL	659	13·2 (11·7–14·3)	13·3 (11·9–14·4)	12·7 (11·1–14·0)	0·001
Creatinine, mg/dL	676	1·4 (1·2–1·8)	1·4 (1·2–1·7)	1·5 (1·2–1·9)	1·000
Sodium, mmol/dL	616	140·0 (138·0–142·0)	141·0 (139·0–142·0)	140·0 (138·0–142·0)	0·294

Continuous variables are median (IQR). Categorical variables are n (%). Missing data are reported as n. ICU=Intensive Care Unit. ED=Emergency Department. CHF=congestive heart failure. COPD=chronic obstructive pulmonary disease. ePA-AC=Elektronische Patientenakte. WBC=white blood cell

### Comparison of original and validation cohorts

Patient characteristics across the development, recalibration, and external validation cohorts is presented in the appendix [see Table S9, Additional file 1]. Patients in the external validation cohort were older (median age 74·0 vs. 67·0 and 71·0 in the development and the recalibration cohorts, respectively) and included fewer medical patients (43·9% vs. 57·4% and 64·4%). The prevalence of dementia, psychiatric disorders, and fractures was lower in the external cohort, whereas history of delirium was higher. Fall risk scores (Hendrich II Fall Risk Model and Hester Davis Scale), rates of opioid use, red blood cell transfusions, and abnormal laboratory values, such as low haemoglobin, elevated white blood cell count, and hyponatraemia, were also generally lower in the external cohort, except for creatinine. Delirium incidence was 17·0% in the validation cohort, compared with 13·8% and 8·1% in the recalibration and development cohorts, respectively. It is important to mention that the age difference in the development and recalibration cohort likely resulted in a validation population with higher baseline delirium risk. The difference in age criteria together with delirium diagnostic methods may influence both delirium incidence and the relationship between predictors and outcome and thus may affect model performance and calibration in this external validation.

### Model performance

The original and recalibrated medical MDP tools were validated on 947 patients (23% experienced delirium), while the tools dedicated to surgical patients were validated on 1212 patients (12% experienced delirium). Validation using ePA-AC fall risk included 751 medical patients (28% developed delirium), and 1054 surgical patients (13% developed delirium). External validation results are reported in Table 2. For medical patients, the original MDP tool achieved higher AUROC across all fall risk scores, with a slight decrease after recalibration. For surgical patients, model performance was broadly comparable between the original and recalibrated tools.

**Fig. 1:**
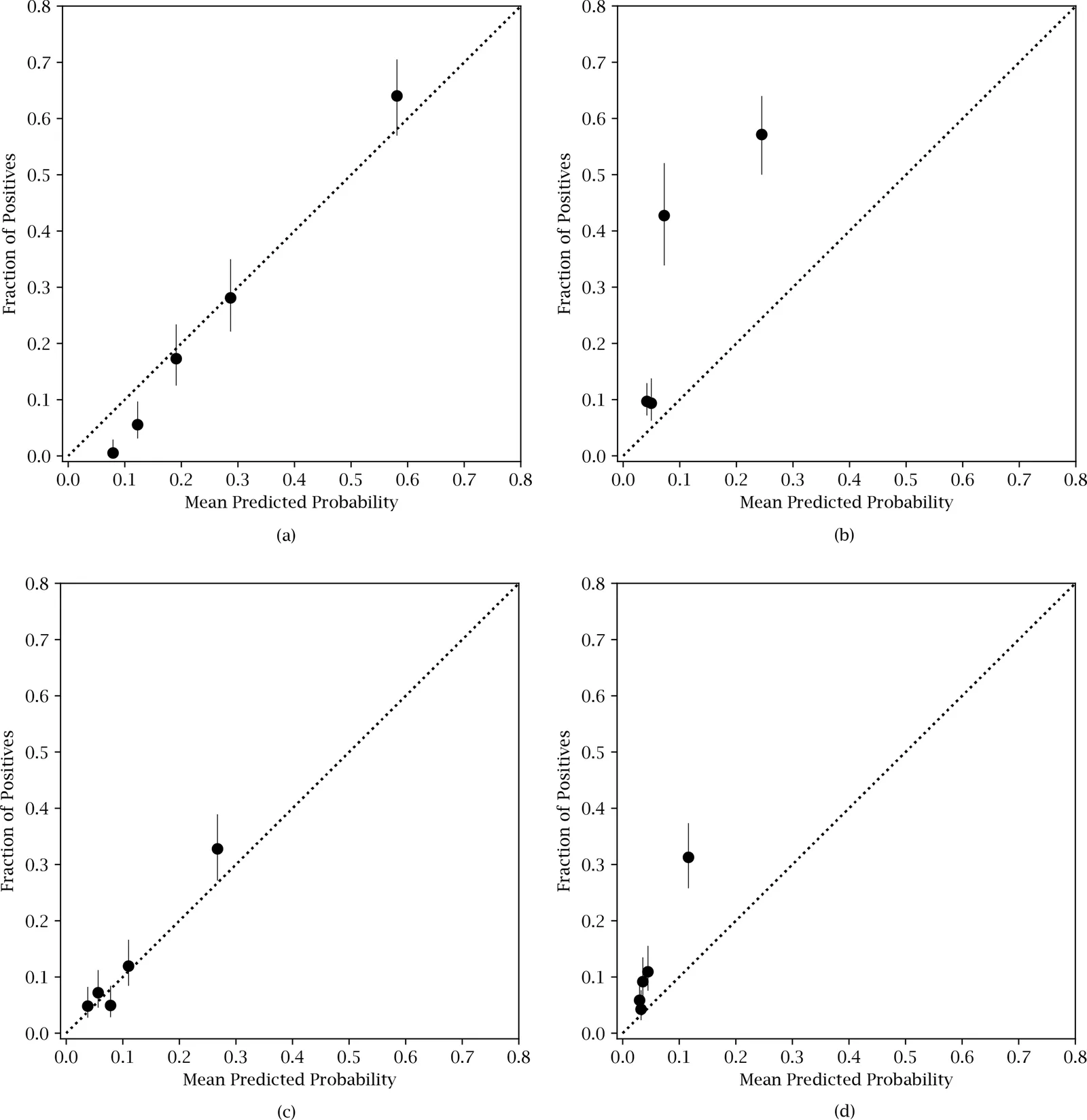
Calibration plots of the original and recalibrated MDP tools using Hester Davis Scale. **(a)** medical MDP tool, original;** (b)** medical MDP tool, recalibrated; **(c) **surgical MDP tool, original; **(d) **surgical MDP tool, recalibrated

Figure 1 depicts the calibration curves and predicted score distributions for models using Hester Davis Scale. Both original medical and surgical MDP tools demonstrated good calibration, whereas recalibrated models consistently underestimated the observed probabilities. Corresponding results for the Hendrich II Fall Risk Model are provided in the appendix [see Figure S1**, **Additional File 1]. The original MDP tool maintained good discrimination across subgroups by sex assigned at birth and age and remained well calibrated when using Hester Davis Scale [see Tables S10–S13, Figures S2–S9, Additional File 1]. It was posited that binarizing the fall risk score reduced its informativeness, thereby negatively impacting both predictive performance and model calibration. In addition, recalibration was performed on a considerably smaller cohort of patients compared to the original development set, which may have limited the stability and generalisability of the recalibrated models. Beyond these methodological factors, differences between the recalibration and validation cohorts may also explain the reduced performance of the recalibrated models. The recalibration cohort was derived from the same hospital as the development cohort but used a different delirium definition and had lower delirium incidence compared to our validation cohort. The recalibrated models may have captured patterns specific to a different diagnostic standard and clinical context, limiting their transportability to our cohort. Nevertheless, predictive performance of the medical MDP tool remains favourable when compared to existing literature.

**Fig. 2:**
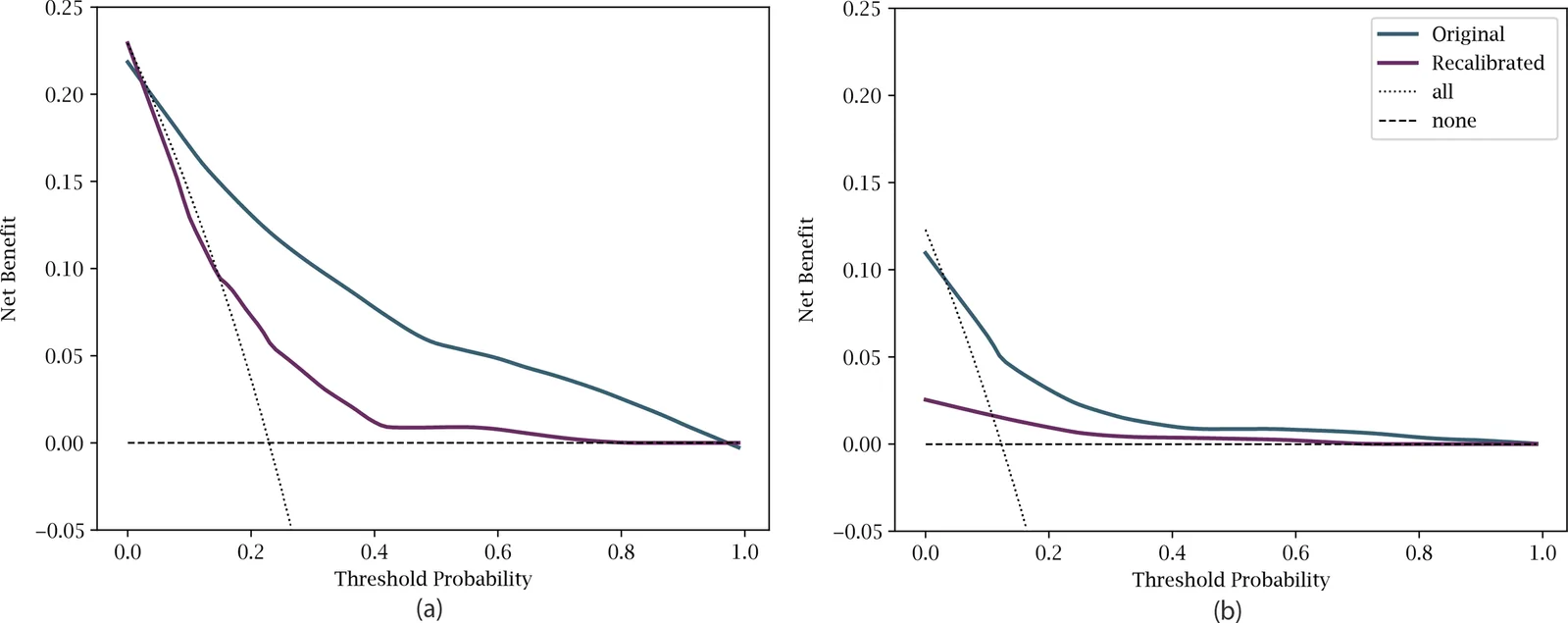
Decision curve analysis (DCA) of the MDP tools using Hester Davis Scale. **(a)** medical MDP tool; **(b)** surgical MDP tool

Figure 2 presents DCA using Hester Davis Scale. In both patient groups, the original models consistently demonstrated higher net benefit across a wide range of clinically relevant threshold probabilities compared to the recalibrated models, indicating better clinical utility for identifying patients at risk of delirium. Moreover, the MDP tools for medical patients showed a consistent overall advantage, while the MDP tools for surgical patients did not provide benefit across most thresholds. Results using the Hendrich II Fall Risk Model are shown in the appendix (Figure S10).

**Table 2:** Predictive performance of the MDP tools using different fall risk scores

Model	Hendrich II Fall Risk Model	Hester Davis Scale	ePA-AC fall risk
Medical – original	0·87 (0·85–0·90)	0·86 (0·83–0·88)	0·82 (0·78–0·85)
Medical – recalibrated	0·78 (0·75–0·82)	0·78 (0·74–0·81)	0·78 (0·74–0·82)
Surgical – original	0·73 (0·69–0·78)	0·74 (0·69–0·79)	0·73 (0·68–0·78)
Surgical – recalibrated	0·72 (0·67–0·76)	0·72 (0·68–0·77)	0·71 (0·66–0·75)

Values are AUROC (95% CI). AUROC=area under the receiver operating characteristic curve. CI=confidence interval

### Sensitivity analysis

The validation excluding any potential sources of data leakage related to the Hester Davis Scale and other diagnoses-related predictors revealed slightly lower performance across all models [see Table S14, Additional file 1**].** This decline was expected, as the attempt to prevent information leakage involved excluding data that may have been valid. Nonetheless, performance of MDP tools for medical patients remained acceptable within the clinical context. An additional validation analysis was conducted on a subset of patients with at least one DOSS measurement recorded per day. This subset included 492 medical patients, of whom 36% developed delirium, and 672 surgical patients, of whom 17% developed delirium. Model performance results are presented in the appendix [see Table S15, Additional file 1]. These findings are consistent with the main analysis, confirming that the MDP tool for medical patients demonstrated stronger discriminative performance compared to the tool for surgical patients. As observed previously, the recalibrated models showed slightly lower performance than their original counterparts.

## Discussion

### Main findings

This single-centre external validation in Swiss hospital patients confirmed that the original MDP tool for medical patients accurately predicts delirium, whereas performance in surgical patients was lower for both the original and recalibrated versions. These results were consistent across sensitivity analyses, including those excluding potential information leakage and those limited to patients with at least one daily delirium assessment. No significant differences in predictive performance were observed among the reconstructed Hester Davis Scale, Hendrich II Fall Risk Model, and ePA-AC fall risk. However, replacing the Hendrich II score with the ePA-AC risk within the original MDP tool reduced predictive performance. This decline was most likely attributable to the use of continuous predictors without any transformation during model training, which made the model sensitive to the value range of continuous variables.

**Fig. 3:**
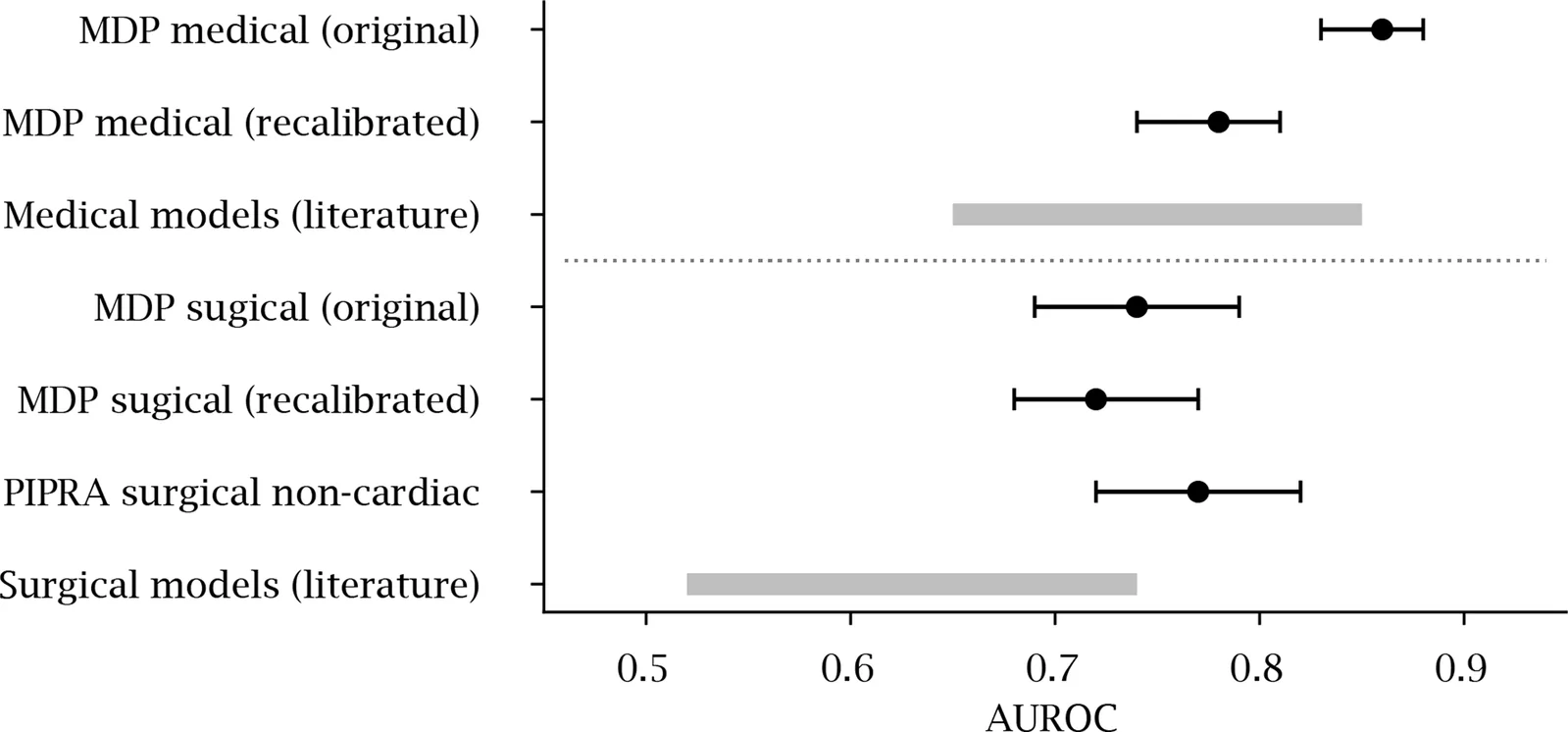
Comparison of the MDP tool with published models Performance of the MDP tool in medical and surgical patients is shown in both original and recalibrated forms. Discrimination is expressed as AUROC (dots) with 95% CIs (horizontal bars). Grey bands indicate the range of AUROC values reported for medical and surgical delirium prediction models in two systematic reviews

Comparison of the MDP tool with existing delirium prediction models help contextualize these findings (Fig. 3**)**. An external validation of 14 delirium prediction models for surgical patients reported AUROCs ranging from 0·52 to 0·74 [21]. In addition, the PIPRA model [22, 23], which has already undergone external validation, is CE-certified, and was developed using a similar logistic regression approach, achieved an AUROC of 0·77 (95% CI: 0·72–0·82) during external validation in non-cardiac surgical population from the same hospital and timeframe [24]. Regarding the medical cohort, a systematic review reported AUROCs ranging from 0·65 to 0·85 [25].

Overall, in this external validation study conducted on Swiss hospital patients, the MDP tool demonstrated good predictive performance in medical patients but limited effectiveness in surgical patients.

### Limitations

This validation study used data from a QIP, therefore no sample size was calculated. In addition, the analyses were constrained by the data structure provided through the QIP. Only aggregated information and limited de-identified extracts linking delirium outcomes to risk scores were available to the research team. While this permitted external validation analyses such as discrimination, calibration, and subgroup assessment, more detailed patient-level or exploratory analyses could not be performed. Inclusion criteria differed from those used in the development and recalibration cohorts, particularly regarding age and the exclusion of individuals with acute substance use disorders.

Delirium was assessed up to seven days postoperatively in the surgical validation cohort, whereas in the development and recalibration cohorts, delirium could be identified at any point during the hospital stay. This discrepancy in follow-up windows may have influenced the observed incidence and estimated model performance.

Delirium diagnosis in the validation cohort was based on DOSS, which differs from the CAM-based tools employed in development and recalibration cohorts. This methodological difference may affect the comparability of results across cohorts and may introduce systematic bias. The observed differences in delirium incidence between cohorts may reflect not only variations in patient populations but also differences in the sensitivity and specificity of the diagnostic tools used.

In the validation cohort, predictors related to specific diagnosis, and the reconstructed Hester Davis Scale may have introduced data leakage. A sensitivity analysis excluding any potential sources of data leakage showed a moderate but acceptable decline in predictive performance.

Race and ethnicity data were unavailable, precluding assessment of model performance across demographic groups. This limits the generalisability of our findings. Future studies should incorporate race/ethnicity data to enhance the robustness and applicability of predictive models.

Finally, a limitation of this study is the presence of missing data for DOSS assessments. Because delirium is frequently underdiagnosed in routine clinical care, it is possible that some episodes were not systematically captured. This limitation might affect the accuracy of the reported delirium incidence.

### Strengths

Despite the limitations, this study presents an extensive external validation of the MDP tool in an independent Swiss cohort. The model maintained robust predictive performance across multiple analyses, despite differences in setting, population, and diagnostic methods. Sensitivity analyses using an alternative fall risk score, excluding potential sources of data leakage, and restricting to patients assessed for delirium at least once daily confirmed the consistency of findings.

As this study was conducted as a QIP rather than a conventional clinical trial, there was no selection bias in patient inclusion. All eligible patients were consecutively included during the study period, effectively removing enrolment bias. This is particularly relevant in delirium research, where the requirement for informed consent in traditional trials can lead to systematic exclusion of vulnerable groups, such as patients with mild cognitive impairment or lower educational backgrounds, who are themselves at higher risk of developing delirium.

### Clinical and research implications

The findings of this study support the potential use of the MDP tool in the healthcare setting for delirium risk stratification. Its performance in an independent cohort, along with its adaptability to different fall risk scores, underscore its generalisability. In particular, the medical MDP tool demonstrated the best performance across all analyses and may be prioritised for implementation in similar patient populations.

Further multicentre validations across diverse health systems and languages are suggested to study the generalisability of the tool. Additionally, future work could explore the integration of the MDP tool into clinical workflow to evaluate its prospective impact in terms of delirium incidence and other secondary outcomes as well as its feasibility as part of automated delirium prevention strategies.

## Conclusion

The MDP tool demonstrated robust predictive performance in an independent European hospital cohort, suggesting potential generalizability beyond the original development setting. These findings warrant multicentre prospective validation to establish effectiveness and clinical impact. 

## Supplementary Information

Below is the link to the electronic supplementary material.

Supplementary Material 1 : Supplementary information on the data. Supplementary tables and figures supporting the model validation results.

## Data Availability

The data supporting the conclusions of this article were collected as part of a hospital QIP and are therefore not available for public sharing, but requests for collaboration are encouraged.

## Abbreviations

AUROC: Area under the receiver operating characteristic curve
CAM-ICU: Confusion Assessment Method for the Intensive Care Unit
CI: Confidence interval
DCA: Decision curve analysis
DOSS: Delirium Observation Screening Scale
DTS: Delirium Triage Screen
ePA-AC: Elektronische Patientenakte
ICD: International Classification of Diseases
IQR: Interquartile range
MDP: Mayo Delirium Prediction tool
QIP: Quality improvement project
US: United States
