# Supplementary material for: Retrospective external validation of the Mayo Delirium Prediction tool in a Swiss cohort of medical and surgical inpatients
Source: BMC Med Inform Decis Mak. 2026 Jun 29;26:351. doi: 10.1186/s12911-026-03660-5 (PMC13579724; doi:10.1186/s12911-026-03660-5)
Supplement: Supplementary file 1 [file 12911_2026_3660_MOESM1_ESM.docx]

**Appendix**

[**Supplemental content 1 – Variables reconstruction process**	2](#_Toc210934959)

[**Sources of variables**	2](#_Toc210934960)

[**Details about the fall risk scores**	4](#_Toc210934961)

[**Reconstructed Variables**	4](#_Toc210934962)

[**Fall risk: Heindrich II Fall Risk Model**	4](#_Toc210934963)

[**Fall risk: Hester Davis Scale**	5](#_Toc210934964)

[**Charlson Comorbidity Index**	7](#_Toc210934965)

[**Supplemental content 2 – Supplemental tables**	8](#_Toc210934966)

[**Supplemental content 2 – Supplemental figures**	10](#_Toc210934967)

# **Supplemental content 1 – Variables reconstruction process**

See separate file for now.

## **Sources of variables**

- Structured data: Directly available as structured data from the electronic health record.
  - Including EPA-AC nursing documentation: structured documentation filled out by the nurses within 24h of hospital stay
- ICD codes: Diagnosis codes linked to the hospital stay
- ATC codes: medication or blood transfusions within the first 24h of hospital stay
- Lab tests within 24h of hospital stay

To avoid bias and to comply with the restrictions from the ethics committee, variables were reconstructed and compiled into the various risk scores automatically and at patient level without supervision. Only the final risk scores were available to the researchers. Researchers and physicians agreed on this harmonisation strategy in advance and did not (and were unable to) alter it retrospectively.

Apart from the diagnosis made on admission, all other diagnoses did not have a timestamp. Since it was unclear when these diagnoses were made, there is a risk of bleed over. This was anticipated and each score was calculated with and without the offending variables. (The diagnosis on admission also did not have a timestamp as such but is collected separately from the other ICD codes and can be assumed to be present from admission.)

**Table S1: Sources of variables**

| **Variable** | **Origin** |
| --- | --- |
| Age | Structured data |
| COPD | ICD code |
| Red blood cell transfusion | ATC code |
| Psychiatric disorder | ICD code |
| ED admission | Structured data |
| Dementia | ICD code |
| ICU admission | Structured data |
| Ulcer (any stage) | ICD code |
| creatinine (>1.5 mg/dl) | Lab |
| Fracture | ICD code |
| Atrial fibrillation | ICD code |
| WBC count (>12x10^9) | Lab |
| Fall risk score | reconstructed as "Heindrich II Fall Risk", "Hester Davis Fall Risk" or used the hospitals own score "epa-AC fall risk" |
| Opioid antagonist | ATC code |
| Charlson scores | reconstructed |
| haemoglobin (<10 g/dl) | Lab |
| History of delirium | Structured data |
| Sodium | Lab |
| Opioid analgesic | ATC code |
| Congestive heart failure | ICD code |

COPD=chronic obstructive pulmonary disease. ICD=International Classification of Diseases. ATC=Anatomical Therapeutic Chemical classification system. ED=emergency department. ICU=intensive care unit. WBC=white blood cell.

**Table S2: ICD codes used**

| Dementia | F01.50, F01.51, F02.80, F02.81, F03.90, F03.91, F05, G30.0, G30.1, G30.8, G30.9, G31.01, G31.09, G31.1, G31.83 |
| --- | --- |
| COPD | J44.0, J44.1, J44.9, J43.0, J43.1, J43.2, J43.8, J43.9, J44.x, J98.2, J98.3, P25.0, P25.8, T79.7XXA, T79.7XXD, T79.7XXS, T81.82XA, T81.82XD, T81.82XS |
| Atrial fibrillation | I48.0, I48.1, I48.2, I48.3, I48.4, I48.91, I48.92 |
| Psychiatric disorder | F23, F24, F25.0, F25.1, F25.8, F25.9, F28, F29, F20.0, F20.1, F20.2, F20.3, F20.5, F20.81, F20.89, F20.9, F84.5, F06.31, F06.32, F25.0, F25.1, F30.10, F30.11, F30.12, F30.13, F30.2, F30.3, F30.4, F30.8, F30.9, F31.0, F31.10, F31.11, F31.12, F31.13, F31.2, F31.30, F31.31, F31.32, F31.4, F31.5, F31.60, F31.61, F31.62, F31.63, F31.64, F31.70, F31.71, F31.72, F31.73, F31.74, F31.75, F31.76, F31.77, F31.78, F31.81, F31.89, F31.9, F32.0, F32.1, F32.2, F32.3, F32.4, F32.5, F32.8, F32.81, F32.89, F32.9, F33.0, F33.1, F33.2, F33.3, F33.40, F33.41, F33.42, F33.8, F33.9, F34.0, F34.1, F34.8, F34.81, F34.89, F34.9, F39, F41.8, F43.21, F43.23, F51.13, F53, O90.6, Z86.59 |
| Neuropathy | G60, G61, G62, G63 |
| ICD: depression | F20.4, F32, F33, F25.1, F34.1, F38.1, F41.2, F53.0 |
| ICD codes: hemiplegic, paraplegia, or quadriplegia | G80.2, G81.0, G81.1, G81.9, G40.1, G11.4, G82, P11.50, P11.51 |
| ICD code substance abuse/alcohol? | F10.1, F10.2, F11.1, F11.2, F12.1, F12.2, F13.1, F13.2, F14.1, F14.2, F15.1, F15.2, F16.1, F16.2, F17.1, F17.2, F18.1, F18.2, F19.1, F19.2 |
| Fracture* *The following fractures were included: head/neck, hip/thigh, knee/ankle/lower leg, spine/pelvis, and thorax. | S02, S12, S72, S82, S32, S12, S22 |
| Ulcer (any stage) | A57, A66.4, A69.0, E50.3, H16.0, I77.8, I83.0, I83.2, I86.88, I87.01, I87.21, J35.8, J38.7, J98.0, K04.0, K12.0, K12.3, K14.0, K22.1, K62.6, K63.3, K82.8, K83.8, L89, L97, L98.4, N77, N34.2, N48.5, N50.8, N76.5, N76.6, N77, N86, N89.8, P78.8 |
| Myocardial infact | I21, I22 |
| CHF (congestive heart failure) | I50.1, I50.20, I50.21, I50.22, I50.23, I50.30, I50.31, I50.32, I50.33, I50.40, I50.41, I50.42, I50.43, I50.8, I50.81, I50.810, I50.811, I50.812, I50.813, I50.814, I50.82, I50.83, I50.84, I50.89, I50.9 |
| peripheral vascular disease | I70.21, I70.22, I70.23, I70.24, I73.0, I73.1, I73.8, I73.9 |
| CVA | I63.30, I63.311, I63.312, I63.319, I63.321, I63.322, I63.329, I63.331, I63.332, I63.339, I63.341, I63.342, I63.349, I63.39, I63.40, I63.411, I63.412, I63.419, I63.421, I63.422, I63.429, I63.431, I63.432, I63.439, I63.441, I63.442, I63.449, I63.49, I63.50, I63.511, I63.512, I63.519, I63.521, I63.522, I63.529, I63.531, I63.532, I63.539, I63.541, I63.542, I63.549, I63.59, G45.9, I69.80, I69.81, I69.820, I69.821, I69.822, I69.823, I69.828, I69.831, I69.832, I69.833, I69.834, I69.839, I69.841, I69.842, I69.843, I69.844, I69.849, I69.851, I69.852, I69.853, I69.854, I69.859, I69.861, I69.862, I69.863, I69.864, I69.865, I69.869, I69.890, I69.891, I69.892, I69.893, I69.898, I69.90, I69.91, I69.920, I69.921, I69.922, I69.923, I69.928, I69.931, I69.932, I69.933, I69.934, I69.939, I69.941, I69.942, I69.943, I69.944, I69.949, I69.951, I69.952, I69.953, I69.954, I69.959, I69.961, I69.962, I69.963, I69.964, I69.965, I69.969, I69.990, I69.991, I69.992, I69.993, I69.998, Z86.73 |
| TIA | G45.9, G45.8, G45.0, G45.1 |
| chronic pulmonary disease | J43, J44 |
| connective tissue disease | M05, M06, M33, M34, M35.0, M35.1, M45 |
| ulcer disease (only linked to the stomach) | K25, K26, K27, K28, K29 |
| mild liver disease | K71.0, K73.0, K73.2, K75.9, K76.0 |
| moderate or severe liver disease | K70.3, K71.7, K72.0, K72.1, K72.9, K74.0, K74.1, K74.2, K74.6 |
| diabetes (without complications) | E10.9, E11.9, E13.9, E14.9 |
| diabetes with end organ damage | E10.2, E11.2, E13.2, E14.2, E10.3, E11.3, E13.3, E14.3, E10.4, E11.4, E13.4, E14.4, E10.5, E11.5, E13.5, E14.5, E10.6, E11.6, E13.6, E14.6, E10.7, E11.7, E13.7, E14.7 |
| diabetes | E08.00, E08.01, E08.10, E08.11, E08.21, E08.22, E08.29, E08.311, E08.319, E08.321, E08.321, E08.3211, E08.3212, E08.3213, E08.3219, E08.329, E08.329, E08.3291, E08.3292, E08.3293, E08.3299, E08.331, E08.331, E08.3311, E08.3312, E08.3313, E08.3319, E08.339, E08.339, E08.3391, E08.3392, E08.3393, E08.3399, E08.341, E08.341, E08.3411, E08.3412, E08.3413, E08.3419, E08.349, E08.349, E08.3491, E08.3492, E08.3493, E08.3499, E08.351, E08.351, E08.3511, E08.3512, E08.3513, E08.3519, E08.352, E08.3521, E08.3522, E08.3523, E08.3529, E08.353, E08.3531, E08.3532, E08.3533, E08.3539, E08.354, E08.3541, E08.3542, E08.3543, E08.3549, E08.355, E08.3551, E08.3552, E08.3553, E08.3559, E08.359, E08.359, E08.3591, E08.3592, E08.3593, E08.3599, E08.36, E08.37, E08.37X1, E08.37X2, E08.37X3, E08.37X9, E08.39, E08.40, E08.41, E08.42, E08.43, E08.44, E08.49, E08.51, E08.52, E08.59, E08.610, E08.618, E08.620, E08.621, E08.622, E08.628, E08.630, E08.638, E08.641, E08.649, E08.65, E08.69, E08.8, E08.9, E09.00, E09.01, E09.10, E09.11, E09.21, E09.22, E09.29, E09.311, E09.319, E09.321, E09.321, E09.3211, E09.3212, E09.3213, E09.3219, E09.329, E09.329, E09.3291, E09.3292, E09.3293, E09.3299, E09.331, E09.331, E09.3311, E09.3312, E09.3313, E09.3319, E09.339, E09.339, E09.3391, E09.3392, E09.3393, E09.3399, E09.341, E09.341, E09.3411, E09.3412, E09.3413, E09.3419, E09.349, E09.349, E09.3491, E09.3492, E09.3493, E09.3499, E09.351, E09.351, E09.3511, E09.3512, E09.3513, E09.3519, E09.352, E09.3521, E09.3522, E09.3523, E09.3529, E09.353, E09.3531, E09.3532, E09.3533, E09.3539, E09.354, E09.3541, E09.3542, E09.3543, E09.3549, E09.355, E09.3551, E09.3552, E09.3553, E09.3559, E09.359, E09.359, E09.3591, E09.3592, E09.3593, E09.3599, E09.36, E09.37, E09.37X1, E09.37X2, E09.37X3, E09.37X9, E09.39, E09.40, E09.41, E09.42, E09.43, E09.44, E09.49, E09.51, E09.52 |
| hemiplegia | G80.2, G81 |
| moderate or severe renal disease | N18.3, N18.4, N18.5, N18.6, N19, N17, Z99.2 |
| Solid tumor localised | C00, C01, C02, C03, C04, C05, C06, C07, C08, C09, C10, C11, C12, C13, C14, C15, C16, C17, C18, C19, C20, C21, C22, C23, C24, C25, C26, C27, C28, C29, C30, C31, C32, C33, C34, C35, C36, C37, C38, C39, C40, C41, C42, C43, C44, C45, C46, C47, C48, C49, C50, C51, C52, C53, C54, C55, C56, C57, C58, C59, C60, C61, C62, C63, C64, C65, C66, C67, C68, C69, C70, C71, C72, C73, C74, C75, C76 |
| Solid tumor metastatic | C77, C78, C79, C80 |
| leukemia | C90.1, C91, C92, C93, C94, C96 |
| Lymphoma | C81, C82, C83, C84, C85, C86, C88.4, C88.0, C91.5 |
| AIDS | B20, B21, B22, B23, B24 |
| Coronary artery disease (CAD) | I25.10, I25.110, I25.111, I25.118, I25.119, I25.2, I25.3, I25.41, I25.42, I25.5, I25.6, I25.700, I25.701, I25.708, I25.709, I25.710, I25.711, I25.718, I25.719, I25.720, I25.721, I25.728, I25.729, I25.730, I25.731, I25.738, I25.739, I25.750, I25.751, I25.758, I25.759, I25.760, I25.761, I25.768, I25.769, I25.790, I25.791, I25.798, I25.799, I25.810, I25.811, I25.812, I25.82, I25.83, I25.84, I25.89, I25.9 |
| CKD (chronic kidney disease) | I12.0, I12.9, I13.0, I13.10, I13.11, I13.2, N18.1, N18.2, N18.3, N18.4, N18.5, N18.6, N18.9, D63.1, E08.22, E09.22, E10.22, E11.22, E13.22 |
| Delirium | F05, F01.51, F02.81, F03.91, F10.221, F10.231, F10.921, F10.96, F10.121, F11.121, F11.221, F11.921, F12.121, F12.221, F12.921, F13.121, F13.221, F13.231, F13.921, F13.931, F14.121, F14.221, F14.921, F15.121, F15.221, F15.921, F16.121, F16.221, F16.921, F18.121, F18.221, F18.921, F19.121, F19.221, F19.231, F19.921, F19.931, R41.0, F43.0 |
| Pneumonia | A02.22, A15.0, A37.01, A37.11, A37.81, A37.91, A50.04, A54.84, B01.2, B05.2, B06.81, B25.0, B58.3, B77.81, J09.X1, J10.00, J10.08, J11.00, J11.08, J12.0, J12.1, J12.2, J12.3, J12.81, J12.89, J12.9, J13, J14, J15.0, J15.1, J15.20, J15.211, J15.212, J15.29, J15.3, J15.4, J15.5, J15.6, J15.7, J15.8, J15.9, J16.0, J16.8, J17, J18.0, J18.1, J18.2, J18.8, J18.9, J67.7, J67.8, J67.9, J68.0, J68.1, J68.2, J68.3, J68.4, J68.8, J68.9, J69.0, J69.1, J69.8, J84.111, J84.113, J84.114, J84.116, J84.117, J84.2, J85.1, J95.4, J95.851, J95.89, O29.011, O29.012, O29.013, O29.019, O74.0, O89.01, P23.0, P23.1, P23.2, P23.3, P23.4, P23.5, P23.6, P23.8, P23.9, Z87.01 |
| Sleep disorder | F51.01, F51.02, F51.03, F51.04, F51.05, F51.09, F51.11, F51.12, F51.13, F51.19, F51.3, F51.4, F51.5, F51.8, F51.9, G47.00, G47.01, G47.09, G47.10, G47.11, G47.12, G47.13, G47.14, G47.19, G47.20, G47.21, G47.22, G47.23, G47.24, G47.25, G47.26, G47.27, G47.29, G47.30, G47.31, G47.32, G47.33, G47.34, G47.35, G47.36, G47.37, G47.39, G47.411, G47.419, G47.421, G47.429, G47.50, G47.51, G47.52, G47.53, G47.54, G47.59, G47.61, G47.62, G47.63, G47.69, G47.8, G47.9 |
| UTI | N30.00, N30.01, N30.10, N30.11, N30.20, N30.21, N30.30, N30.31, N30.40, N30.41, N30.80, N30.81, N30.90, N30.91, N39.0, B37.41, B37.42, B37.49, P39.3, A54.01, A56.01, A59.03, N34.0, N34.1, N34.2, N34.3 |

COPD=chronic obstructive pulmonary disease. ICD=International Classification of Diseases. CHF congestive heart failure. CVA=cerebrovascular accident. TIA=transient ischaemic attack. AIDS=acquired immunodeficiency syndrome. CKD=chronic kidney disease. UTI=urinary tract infection.

**Table S3: ATC codes used**

| Non-narctoic analgesics | N02B |
| --- | --- |
| Proton pump inhibitors | A02BC |
| Opioid analgesics | N02A |
| Opioid antagonist | A06AH |
| Sedatives | N05C |
| Anti-depressant | N06A |
| Anesthetic | N01 |
| Benzodiazepines | N05BA |
| CNS stimulant | CN800, CN809 |
| Steroid | H02 |
| Anti-Epileptic | N03 |
| Anti-cholinergic | N04A, G04BD |
| Anti-psychotic | N05A |
| Opioid antagonist | A06AH |
| Muscle relaxant | MO3 |
| Anti-Parkinson | N04 |
| Chemotherapy | L01 |
| Anti-dementia | N06D |
| CV meds | C01, C02, C03, C04, C05, C07, C08, C09, C10 |
| CNS meds | N01, N02, N03, N04, N05, N06, N07 |
| Diuretics | C03 |
| Blood products (transfusion) | B05AX01, B05AX02, B05AX03 |

CNS=central nervous system. CV=cardiovascular.

## **Details about the fall risk scores**

The Hendrich II Fall Risk Model is a widely used screening tool in acute care hospitals in the United States, and it consists of a small set of risk factors, including confusion, depression, and elimination patterns, resulting in a numerical score (score range zero to 16). In contrast, the Hester Davis Scale provides a more comprehensive assessment, combining a broader range of factors such as age, medication classes, use of assistive devices, mobility status, and cognitive function. This scale is also commonly used in United States hospitals and allows for finer stratification of fall risk (score range zero to 25+). The ePA-AC fall risk is a structured, nursing assessment routinely embedded within the electronic nursing documentation systems of German, Austrian and Swiss hospitals. While its structure may vary slightly between institutions, it typically includes elements such as mobility, cognitive status, toileting needs, and prior fall history (score range zero to three).

## **Reconstructed Variables**

### **Fall risk: Heindrich II Fall Risk Model**

To reconstruct the Hendrich II Fall Risk Model, points were given when a condition was true and the sum of all points is the reconstructed Heindrich II fall risk. In the reconstruction table, the logical operator “OR” is used in the sense of **“and/or”,** meaning that the presence of any one or a combination of the listed conditions is sufficient to meet the criterion.

**Table S4: Hendrich II Fall Risk Model**

| **Risk factor** | **Points** | **Variables used (original)** | **English translation** |
| --- | --- | --- | --- |
| Confusion/disorientation/impulsivity | 4 | Aufmerksamkeit <>"nicht beeintraechtigt" OR Orientierung <> "zu allen Qualitaeten" OR Merkmale Herausforderndes Verhalten <> "Nein" OR Abklaerungserfordernis Verwirrung/Demenz/Delir <> "Keine" | Attention <>"not impaired" OR Orientation <> "to all qualities" OR Characteristics Challenging Behavior <> "No" OR Need for Assessment Confusion/Dementia/Delirium <> "None" |
| symptomatic depression | 2 | ICD code for Depression AND Traurigkeit<>"keine" | ICD code for Depression AND Sadness<>"none" |
| altered elimination | 1 | SBF Urinausscheidung <> "volle Fähigkeit" OR Urinausscheidung kontrolliert <> "vollständige Kontrolle" OR SBF Stuhlausscheidung <> "volle Fähigkeit" OR Stuhlausscheidung kontrolliert<>"vollständige Kontrolle" OR Dranghafte/ gesteigerte Ausscheidung <> "Nein" | SBF Urine output <> "full capacity" OR Urine output controlled <> "full control" OR SBF Stool output <> "full capacity" OR Stool output controlled <> "full control" OR Urgent/increased elimination <> "No" |
| dizziness/vertigo | 1 | Gleichgewichtsstoerung <> "nein" | Balance disorder <> "no" |
| gender (male) | 1 | structured data | structured data |
| Antiepileptics | 2 | ATC codes | ATC codes |
| Benzodiazepines | 1 | ATC codes | ATC codes |
| **get up and go test "rising from a chair"** | | | |
| Ability to rise in single movement - no loss of balance with steps | 0 | SPF Fortbewegung = volle Fähigkeit | SPF movement = full ability |
| pushes up, successful in one attempt | 1 | SPF Fortbewegung = gering beeinträchtigt | SPF movement = slightly impaired |
| multiple attempts but successful | 3 | SPF Fortbewegung = stark beeinträchtigt | SPF movement = severely impaired |
| unable to rise without assistance during test | 4 | SPF Fortbewegung = keine Fähigkeit/Bettruhe | SPF movement = no ability/bed rest |

ICD=International Classification of Diseases. ATC=Anatomical Therapeutic Chemical classification system. SPF= structured physical function.

### **Fall risk: Hester Davis Scale**

The Hester Davis Scale was reconstructed based on the original scale definitions. Each domain (e.g., age, mobility, medication) contains multiple possible conditions, each associated with a predefined point value (range 0–4). For scoring, a single value was assigned per domain according to the following rules: (i) only one condition per domain was counted, (ii) if multiple conditions were present, the condition with the highest corresponding point value was selected, and (iii) the total reconstructed Hester Davis Scale was calculated as the sum of all domain scores. In the reconstruction table, the logical operator “OR” is used in the sense of **“and/or”,** meaning that the presence of any one or a combination of the listed conditions is sufficient to meet the criterion.

**Table S5: Hester Davis Scale**

| **Category** | **Description** | **Points** | **Variables used (original)** | **English translation** |
| --- | --- | --- | --- | --- |
| Age | Age <60 | 2 | Age |  |
|  | Age >60 | 3 | Age |  |
| last known fall | no fall | 0 | default |  |
|  | within last year | 1 | Sturzvorgeschichte="Ja" | History of falls="Yes" |
|  | within last 6 months | 2 |  |  |
|  | within last month | 3 |  |  |
|  | during the current hospitalisation | 4 | aktuelles Sturzereignis = "Ja" | current fall event = "Yes" |
| mobility | no limitation | 0 | default | default |
|  | Dizziness/generalised weakness | 1 | SPF Fortbewegung <>"volle Fähigkeit" OR Gleichgewichtsstoerung="Ja" | SPF Movement <>"full ability" OR Balance impairment="Yes" |
|  | immobilized/requires assist of one person | 2 | veraendertes Gangbild="Ja" | altered gait="Yes" |
|  | use of assistive device/requires assist of two people | 3 | Hilfsmittel Fortbewegung="Ja" | Aids for movement="Yes" |
|  | hemiplegic, paraplegia, or quadriplegia | 4 | ICD codes | ICD codes |
| Medication | no meds | 0 | default | default |
|  | CV or CNS meds | 1 | ATC codes | ATC codes |
|  | CV and CNS meds | 2 | ATC codes | ATC codes |
|  | Diuretics | 3 | ATC codes | ATC codes |
|  | chemotherapy in the last month | 4 | ATC codes | ATC codes |
| mental status/loc/awareness | A, A, Ox3 | 0 | default | default |
|  | Oriented to person and place | 1 | Orientierung = "zu drei Qualitäten" | Orientation = "to three qualities" |
|  | Lethargic/oriented to person only | 2 | Orientierung = "zu einer oder zwei Qualitäten" | Orientation = "to one or two qualities" |
|  | Memory loss/confusion and requires reorienting | 3 | Orientierung = "zu keiner Qualität" OR Kentnisse erwerben = "stark beeinträchtigt" | Orientation = "no quality" OR Acquiring knowledge = "severely impaired" |
|  | Unresponsiveness/noncompliance with instruction | 4 | Bewusstsein="somnolent" OR Bewusstsein="soporös" OR herausfordernden verhalten <>"Nein" | Consciousness="somnolent" OR Consciousness="soporous" OR challenging behavior <>"No" |
| Toileting needs | No needs | 0 | default | default |
|  | Use of catheters or diversion devices | 1 | Urinableitungssystem="Ja" OR Stuhlableitungssystem="Ja" | Urine drainage system="Yes" OR Stool drainage system="Yes" |
|  | use of assistive devices (BSC, bedpan, urinal) | 2 | SBF Fortbewegung = "stark beeinträchtigt" oder SBF Fortbewegung ="keine Fähigkeit/Bettruhe" | SBF movement = "severely impaired" or SBF movement = "no ability/bed rest" |
|  | Incontinence | 3 | SBF Urinausscheidung = "keine Fähigkeit" OR Urinausscheidung kontrolliert = "keine Fähigkeit" OR SBF Stuhlausscheidung = "keine Fähigkeit" OR Stuhlausscheidung kontrolliert = "keine Fähigkeit" | SBF Urine output = "no ability" OR Urine output controlled = "no ability" OR SBF Stool output = "no ability" OR Stool output controlled = "no ability" |
|  | Diarrhea/frequency/urgency | 4 | Drangh. Ausscheidung = "Ja" | Urgent excretion = "Yes" |
| Volume/electrolyte status | no problems | 0 | default | default |
|  | NPO >24h | 1 | Nahrungsmenge oral ="Keine" | Amount of food orally ="None" |
|  | Use of IV fluids/tube feeds | 2 | Sondenernaehrung="Ja" OR Fluessigkeitsergaenzung<>"0 ml (keine Ergänzung)" | Tube feeding="Yes" OR Fluid supplementation<>"0 ml (no supplementation)" |
|  | Nausea/vomiting | 3 | Uebelkeit="Übelkeit (Fremdeinschätzung)" OR Uebelkeit="Übelkeit (Selbsteinschätzung)" | Nausea="Nausea (external assessment)" OR Nausea="Nausea (self-assessment)" |
|  | low blood sugar/electolyte imbalance | 4 | any of these outside normal range defined by the EHR: Natrium, Kalium, Calcium. OR Low Glucose (serum) below range | any of these outside normal range defined by the EHR: sodium, potassium, calcium. OR Low Glucose (serum) below range |
| communication/sensory | no deficits | 0 | default | default |
|  | visual (glasses)/hearing deficit | 1 | Hilfsmittel Hoeren="Ja" OR Hilfsmittel Sehen="Ja" OR Hoeren <>"keine Hörbeeinträchtigung", Sehen<>"keine Sehbeeinträchtigung" | Aids Hearing="Yes" OR Aids Vision="Yes" OR Hearing <>"no hearing impairment", Vision <>"no visual impairment" |
|  | non-english pt/unable to speak/slurred speech | 2 | Fremdsprachigkeit = "Ja", sich mitteilen <>"volle Fähigkeit" | Foreign language skills = "Yes", communicating <>"full ability" |
|  | neuropathy | 3 | ICD codes | ICD codes |
|  | blindness or recent visual change | 4 | Sehen ="sieht nichts/Blindheit" | Seeing = "sees nothing/blindness" |
| behavioral | appropriate behaviour | 0 | default | default |
|  | depression/anxiety | 1 | Angst = "S: mittelstarke Angst (VAS 4-6)" OR Angst = "S: starke Angst (VAS 7-10)" OR Traurigkeit="S: mittelstarke Traurigkeit (VAS 4-6)" OR Traurigkeit="S: starke Traurigkeit (VAS 7-10)" | Anxiety = "S: moderate anxiety (VAS 4-6)" OR Anxiety = "S: severe anxiety (VAS 7-10)" OR Sadness = "S: moderate sadness (VAS 4-6)" OR Sadness = "S: severe sadness (VAS 7-10)" |
|  | behavioural noncompliance with instructions | 2 | herausfordernden verhaltens <> "Nein" | challenging behavior <> "No" |
|  | ETOH/substance abuse | 3 | ICD codes | ICD codes |
|  | Impulsiveness | 4 | herausfordernden verhaltens ="agitiert nicht aggressives Verhalten (körperl./verbal)" OR herausfordernden verhaltens = "kombiniert aggressives und nicht aggresives agitiertes Verhalten" | challenging behavior = "agitates non-aggressive behavior (physical/verbal)" OR challenging behavior = "combines aggressive and non-aggressive agitated behavior" |

ICD=International Classification of Diseases. CV=cardiovascular. CNS=central nervous system. ATC=Anatomical Therapeutic Chemical classification system. BSC=bedside commode. SBF=structured bodily function. SPF=structured physical function. NPO=nil per os. IV=intravenous. EHR=electronic health record. VAS=visual analogue scale. ETOH=ethyl alcohol.

### **Charlson Comorbidity Index**

**Table S6: Charlson Comorbidity Index**

| **Points** | **Category** |
| --- | --- |
| 2 | Aged 60-69 |
| 3 | Aged 70-79 |
| 4 | Aged 80+ |
| 1 | Myocardial infarct |
| 1 | CHF (congestive heart failure) |
| 1 | peripheral vascular disease |
| 1 | CVA or TIA |
| 1 | Dementia |
| 1 | chronic pulmonary disease |
| 1 | connective tissue disease |
| 1 | ulcer disease |
| 1 | mild liver disease |
| 3 | moderate or severe liver disease |
| 1 | diabetes (without complications) |
| 2 | diabetes with end organ damage |
| 2 | hemiplegia |
| 2 | moderate or severe renal disease |
| 2 | Solid tumour localised |
| 6 | Solid tumour metastatic |
| 2 | leukaemia |
| 2 | Lymphoma |
| 6 | AIDS |

CHF=congestive heart failure. CVA=cerebrovascular accident. TIA=transient ischaemic attack. AIDS=acquired immunodeficiency syndrome.
**Supplemental content 2 – Supplemental tables**

**Table S7: Variables collected during the QIP study**

| **Type** | **Predictors** |
| --- | --- |
| **Demographics** | Age; sex assigned at birth; body mass index (BMI) |
| **Admission type** | Medical or surgical admission; Emergency Department (ED) admission; Intensive Care Unit (ICU) admission |
| **Comorbidities** | Cognitive impairment; history of delirium; vitamin D deficiency (VDD); hemiplegia/paraplegia/quadriplegia; incontinence; diarrhoea; nausea/vomiting; low blood sugar; electrolyte imbalance; neuropathy; depression; anxiety; alcohol or substance-use disorder; congestive heart failure (CHF); fracture; ulcer (any stage); dementia; chronic obstructive pulmonary disease (COPD); atrial fibrillation (AFib); Psychiatric disorder |
| **Laboratory variables** | white blood cell (WBC) count; haemoglobin; creatinine; sodium; C-reactive protein (CRP) |
| **Medications** | Antiepileptics; benzodiazepines; opioid analgesic; opioid antagonist; number of medications; cardiovascular (CV) medications; central nervous system (CNS) medications; diuretics; chemotherapy in the last month |
| **Scores** | Surgery risk; American Society of Anesthesiologists Physical Status Classification (ASA); ePA-AC fall risk; Hendrich II Fall Risk Model; Hester Davis Scale; DOSS; Charlson Comorbidity Index, |
| **Others** | Laparotomy/thoracotomy; red blood cell transfusion; use of catheters or diversion devices; use of assistive devices (bedpan, urinal, etc); nil per os (NPO) >24 h; use of intravenous (IV) fluids or tube feeds |

**Mayo Delirium Prediction tools**

The original MDP tool was obtained using Least Absolute Shrinkage and Selection Operator (LASSO) penalized logistic regression, separately for medical and surgical patients, with predictors selected based on the contextual literature. The tool incorporated the Hendrich II Fall Risk Model as a continuous predictor. In the external validation and recalibration study, the fall risk was dichotomised, and two different fall risk scores were considered. The Hendrich II Fall Risk Model was categorized into high risk ≥ five vs. low risk < five, and the Hester Davis Scale was categorised into high risk at a threshold ≥ 13. Recalibration was performed with LASSO, retaining the original MDP tool predictors except that the Hendrich II Fall Risk Model was substituted by the Hester Davis Scale. **Table S8** includes the odds ratio (OR) of the four models.

**Table S8: Odds ratio (OR) estimates for predictors included in the original and recalibrated MDP tools**

| **Variable** | **Medical – original** | **Medical – recalibrated** | **Surgical – original** | **Surgical – recalibrated** |
| --- | --- | --- | --- | --- |
| Age | ·· | ·· | 1·02 | 1·01 |
| COPD | ·· | ·· | 1·02 | 1·52 |
| Red blood cell transfusion | ·· | ·· | 1·13 | 1·42 |
| Psychiatric disorder | ·· | 1·14 | 1·27 | - |
| ED admission (not elective) | ·· | ·· | 1·62 | 1·95 |
| Dementia | 2·16 | 2·20 | 3·49 | 2·23 |
| ICU admission | 2·51 | 4·31 | 3·49 | 3·6 |
| Ulcer (any stage) | 1.55 | ·· | 1.99 | ·· |
| Creatinine (>1·5 mg/dL) | 1·13 | 1·20 | 1·6 | 1·32 |
| Fracture | 1·26 | ·· | 1·36 | ·· |
| Atrial fibrillation | 1·07 | ·· | 1·3 | 1·07 |
| WBC count (>12x10^9 cell/Liter) | 1·05 | 1·27 | 1·2 | 1·43 |
| Fall risk score | 1·23 | 4·06 | 1·14 | 2·18 |
| Narcotic antagonist | 1·09 | ·· | 1·11 | ·· |
| Charlson Comorbidity Index | 1·02 | ·· | 1·07 | ·· |
| Haemoglobin (<10 g/dL) | 1·06 | ·· | 1·04 | 1·46 |
| History of delirium | 1·46 | 1·36 | ·· | ·· |
| Sodium (<130 mmol/dL) | 1·15 | ·· | ·· | ·· |
| Narcotic analgesic | 1·02 | ·· | ·· | ·· |
| CHF | 1·01 | ·· | ·· | ·· |

COPD=chronic obstructive pulmonary disease. ED=emergency department. ICU=Intensive Care Unit. WBC=white blood cell count. CHF=congestive heart failure.

**Table S9: Comparison of patient populations across development, recalibration, and validation cohorts**

|  | **Development & internal validation cohort (n=120764)** | **Recalibration cohort**  **(n=8055)** | **External validation cohort**  **(n=2159)** |
| --- | --- | --- | --- |
| Age, years, mean (SD) | 68·0 (10·9) | 71·8 (11·5) | 74·4 (8·5) |
| Age, years | 67·0 (59·0–76·0) | 71·0 (··) | 74·0 (67·0–81·0) |
| Female | 54802 (45·4%) | 3643 (45·2%) | 953 (44·2%) |
| Medical patients | 69294 (57·4%) | 5187 (64·4%) | 947 (43·9%) |
| ICU admission | 26303 (21·8%) | 1836 (22·8%) | 133 (6·2%) |
| ED admission | 31580 (26·2%) | 2868 (35·6%) | 602 (27·9%) |
| Dementia | 8853 (7·3%) | 968 (12·0%) | 115 (5·3%) |
| CHF | 22301 (18·5%) | 2740 (34·0%) | 234 (10·8%) |
| Atrial Fibrillation | 25055 (20·8%) | 2538 (31·5%) | 440 (20·4%) |
| History of Delirium | 2558 (2·1%) | ·· | 45 (4·8%) |
| Fracture | 4743 (3·9%) | 493 (6·1%) | 52 (2·4%) |
| Ulcer (any stage) | 2157 (1·8%) | 106 (1·3%) | 52 (2·4%) |
| Psychiatric disorder | 70577 (58·4%) | 6210 (77·1%) | 95 (4·4%) |
| COPD | 17074 (14·1%) | 1871 (23·2%) | 36 (1·7%) |
| Opioid analgesic | 89511 (74·1%) | 4870 (60·5%) | 348 (16·1%) |
| Opioid antagonist | 20167 (16·7%) | 145 (1·8%) | 0 (0·0%) |
| Red blood cell transfusion | 12268 (10·2%) | 468 (5·8%) | 10 (0·5%) |
| Hendrich II Fall Risk Model | 2·0 (1·0–4·0) | ·· | 1·0 (1·0–3·0) |
| High fall risk (Heindrich II) | ·· | ·· | 257 (12·7%) |
| Hester Davis Scale | ·· | ·· | 8·0 (5·0–10·0) |
| High fall risk (Hester Davis) | ·· | 2562 (31·8%) | 248 (11·5%) |
| Charlson Comorbidity Index | 5·0 (3·0–7·0) | 9·0 (··) | 4·0 (3·0–5·0) |
| WBC count >12 × 10^9^ cell/L | 27080 (25·9%) | 1960 (27·7%) | 168 (7·8%) |
| Haemoglobin <10 g/dL | 22224 (21·3%) | 1397 (19·8%) | 140 (6·5%) |
| Creatinine >1·5 mg/dL | 12338 (12·1%) | 1499 (21·0%) | 669 (31·0%) |
| Sodium < 130 mmol/dL | 3264 (3·4%) | 165 (2·3%) | 36 (1·7%) |
| Delirium | 9738 (8·1%) | 1107 (13·8%) | 366 (17·0%) |

Continuous variables are median (IQR), unless otherwise specified. Categorical variables are n (%). ICU=Intensive Care Unit. ED=Emergency Department. CHF=congestive heart failure. COPD=chronic obstructive pulmonary disease. ePA-AC=Elektronische Patientenakte. WBC=white blood cell.

**Table S10: Predictive performance of models within patient subgroups stratified by sex assigned at birth using Hester Davis Scale**

| **Model** | **Male** | **Female** |
| --- | --- | --- |
| Medical – original | 0·84 (95% CI 0·80–0·88) | 0·87 (95% CI 0·83–0·90) |
| Medical – recalibrated | 0·80 (95% CI 0·75–0·85) | 0·77 (95% CI 0·71–0·83) |
| Surgical – original | 0·75 (95% CI 0·68–0·81) | 0·72 (95% CI 0·65–0·80) |
| Surgical – recalibrated | 0·72 (95% CI 0·66–0·78) | 0·73 (95% CI 0·65–0·80) |

Performance is reported as AUROC (95% CI). AUROC=area under the receiver operating characteristic curve. CI=confidence interval.

**Table S11: Predictive performance of models within patient subgroups stratified by age using Hester Davis Scale**

| **Model** | **Age ≤ 75** | **Age > 75** |
| --- | --- | --- |
| Medical – original | 0·86 (95% CI 0·80–0·92) | 0·82 (95% CI 0·78–0·86) |
| Medical – recalibrated | 0·77 (95% CI 0·68–0·85) | 0·77 (95% CI 0·72–0·81) |
| Surgical – original | 0·68 (95% CI 0·61–0·75) | 0·76 (95% CI 0·69–0·82) |
| Surgical – recalibrated | 0·67 (95% CI 0·59–0·74) | 0·74 (95% CI 0·68–0·80) |

Performance is reported as AUROC (95% CI). AUROC=area under the receiver operating characteristic curve. CI=confidence interval.

**Table S12: Predictive performance of models within patient subgroups stratified by sex assigned at birth using Hendrich II Fall Risk Model**

| **Model** | **Male** | **Female** |
| --- | --- | --- |
| Medical – original | 0·89 (0·86–0·92) | 0·87 (0·82–0·91) |
| Medical – recalibrated | 0·81 (0·76–0·86) | 0·76 (0·70–0·81) |
| Surgical – original | 0·74 (0·67–0·80) | 0·73 (0·66–0·80) |
| Surgical – recalibrated | 0·72 (0·66–0·78) | 0·71 (0·64–0·78) |

Performance is reported as AUROC (95% CI). AUROC=area under the receiver operating characteristic curve. CI=confidence interval.

**Table S13: Predictive performance of models within patient subgroups stratified by age using Hendrich II Fall Risk Model**

| **Model** | **Age ≤ 75** | **Age > 75** |
| --- | --- | --- |
| Medical – original | 0·86 (0·78–0·92) | 0·86 (0·83–0·90) |
| Medical – recalibrated | 0·78 (0·69–0·86) | 0·77 (0·73–0·82) |
| Surgical – original | 0·69 (0·61–0·76) | 0·74 (0·68–0·81) |
| Surgical – recalibrated | 0·67 (0·59–0·74) | 0·72 (0·66–0·78) |

Performance is reported as AUROC (95% CI). AUROC=area under the receiver operating characteristic curve. CI=confidence interval.

**Table S14: Predictive performance of the MDP tools after exclusion of data leakage**

| **Model** | **Hendrich II Fall Risk Model** | **Hester Davis Scale** |
| --- | --- | --- |
| Medical – original | 0·83 (0·80–0·86) | 0·81 (0·78–0·84) |
| Medical – recalibrated | 0·73 (0·69–0·76) | 0·72 (0·68–0·76) |
| Surgical – original | 0·68 (0·63–0·73) | 0·67 (0·64–0·74) |
| Surgical – recalibrated | 0·66 (0·61–0·71) | 0·67 (0·63–0·73) |

Performance is reported as AUROC (95% CI). AUROC=area under the receiver operating characteristic curve. CI=confidence interval.

**Table S15: Predictive performance of the model in patients screened for delirium at least once per day**

| **Model** | **Hendrich II Fall Risk Model** | **Hester Davis Scale** |
| --- | --- | --- |
| Medical – original | 0·88 (0·85–0·91) | 0·83 (0·80–0·87) |
| Medical – recalibrated | 0·80 (0·76–0·84) | 0·79 (0·74–0·83) |
| Surgical – original | 0·73 (0·67–0·78) | 0·72 (0·66–0·77) |
| Surgical – recalibrated | 0·70 (0·65–0·76) | 0·71 (0·65–0·77) |

Performance is reported as AUROC (95% CI). AUROC=area under the receiver operating characteristic curve. CI=confidence interval.

# **Supplemental content 2 – Supplemental figures**

**Figure S1: Calibration plots of the original and recalibrated MDP tools using Hendrich II Fall Risk Model**

(a) medical MDP tool, original; (b) medical MDP tool, recalibrated; (c) surgical MDP tool, original; (d) surgical MDP tool, recalibrated.

**Figure S2: Calibration curves for the medical MDP tool stratified by sex assigned at birth using Hester Davis Scale**

(a) original MDP tool in male patients; (b) original MDP tool in female patients; (c) recalibrated MDP tool in male patients; (d) recalibrated MDP tool in female patients*.*

**Figure S3: Calibration curves for the medical MDP tool stratified by sex assigned at birth using Hendrich II Fall Risk Model**

(a) original MDP tool in male patients; (b) original MDP tool in female patients; (c) recalibrated MDP tool in male patients; (d) recalibrated MDP tool in female patients*.*

**Figure S4: Calibration curves for the surgical MDP tool stratified by sex assigned at birth using Hester Davis Scale**

(a) original MDP tool in male patients; (b) original MDP tool in female patients; (c) recalibrated MDP tool in male patients; (d) recalibrated MDP tool in female patients.

**Figure S5:** **Calibration curves for the surgical MDP tool stratified by sex assigned at birth using Hendrich II Fall Risk Model**

(a) original MDP tool in male patients; (b) original MDP tool in female patients; (c) recalibrated MDP tool in male patients; (d) recalibrated MDP tool in female patients.

**Figure S6: Calibration curves for the medical MDP tool stratified by age group using Hester Davis Scale**

(a) original MDP tool in patients aged ≤75 years; (b) original MDP tool in patients aged >75 years; (c) recalibrated MDP tool in patients aged ≤75 years; (d) recalibrated MDP tool in patients aged >75 years.

**Figure S7:** **Calibration curves for the medical MDP tool stratified by age group using Hendrich Fall Risk Scale**

(a) original MDP tool in patients aged ≤75 years; (b) original MDP tool in patients aged >75 years; (c) recalibrated MDP tool in patients aged ≤75 years; (d) recalibrated MDP tool in patients aged >75 years.

**Figure S8:** **Calibration curves for the surgical MDP tool stratified by age group using Hester Davis Scale**

(a) original MDP tool in patients aged ≤75 years; (b) original MDP tool in patients aged >75 years; (c) recalibrated MDP tool in patients aged ≤75 years; (d) recalibrated MDP tool in patients aged >75 years.

**Figure S9:** **Calibration curves for the surgical MDP tool stratified by age group using Hendrich II Fall Risk Model**

(a) original MDP tool in patients aged ≤75 years; (b) original MDP tool in patients aged >75 years; (c) recalibrated MDP tool in patients aged ≤75 years; (d) recalibrated MDP tool in patients aged >75 years.

**Figure S10: Decision curve analysis (DCA) of the MDP tools using Hendrich II Fall Risk Model**

(a) medical MDP tool; (b) surgical MDP tool.
